# Engineering biomimetic nanovesicles for PEBP1 mRNA delivery to inhibit ferroptosis in abdominal aortic aneurysm

**DOI:** 10.1002/btm2.70025

**Published:** 2025-07-21

**Authors:** Lulu Chen, Bicheng Chen, Xiang Su

**Affiliations:** ^1^ Department of Anesthesiology The First Affiliated Hospital of Wenzhou Medical University Wenzhou PR China; ^2^ Key Laboratory of Diagnosis and Treatment of Severe Hepato‐Pancreatic Diseases of Zhejiang Province The First Affiliated Hospital of Wenzhou Medical University Wenzhou PR China; ^3^ Department of Vascular Surgery The First Affiliated Hospital of Wenzhou Medical University Wenzhou PR China

**Keywords:** abdominal aortic aneurysm, biomimetic nanovesicles, ferroptosis, NRF2/GPX4 axis, PEBP1, vascular smooth muscle cells

## Abstract

An abdominal aortic aneurysm (AAA) is a life‐threatening vascular condition characterized by the dilation of the abdominal aorta, with ferroptosis playing a significant role in its pathogenesis. This study investigates the therapeutic potential of engineering biomimetic nanovesicles to deliver phosphatidylethanolamine‐binding protein 1 (PEBP1) mRNA for inhibiting ferroptosis in vascular smooth muscle cells (VSMCs) and preventing AAA progression. Differential gene expression analysis of the AAA transcriptomic dataset GSE57691 identified 243 differentially expressed genes (DEGs), intersecting with 12 ferroptosis‐related genes. Single‐cell analysis of dataset GSE237230 highlighted PEBP1 as a key gene in VSMCs. Overexpression of PEBP1 in VSMCs enhanced proliferation, reduced reactive oxygen species (ROS) and iron levels, and inhibited apoptosis and ferroptosis via the NRF2/GPX4 axis. The engineered biomimetic nanovesicles demonstrated significant uptake by VSMCs and effective delivery of PEBP1 mRNA. In vivo studies confirmed that these nanovesicles substantially inhibited AAA progression in mice. This study presents a novel bioengineering approach for AAA treatment by targeting ferroptosis through PEBP1 mRNA delivery, offering a promising molecular strategy for the prevention and management of AAA.

**Translational Impact Statement**

Translational Impact Statement

This study presents a novel therapeutic strategy for abdominal aortic aneurysm (AAA) by leveraging engineered biomimetic nanovesicles to deliver PEBP1 mRNA into vascular smooth muscle cells (VSMCs), thereby activating the NRF2/GPX4 antioxidant axis and inhibiting ferroptosis. Through comprehensive in vitro and in vivo evaluations, the approach demonstrates the ability to significantly reduce oxidative stress, cellular apoptosis, and aneurysm progression. These findings offer a promising foundation for the development of molecular‐targeted therapies utilizing mRNA‐loaded nanovesicles for vascular diseases like AAA.

## 1 INTRODUCTION

Abdominal aortic aneurysm (AAA) is a common and deadly vascular disease characterized by localized dilation of the abdominal aorta. If left untreated, it may lead to fatal arterial rupture.^1^, ^2^, ^3^ The incidence of AAA significantly increases with age, with a higher prevalence in males than in females.^4^, ^5^, ^6^ This condition not only severely impacts patients' quality of life but also imposes a substantial healthcare burden.^7^, ^8^ Despite current treatment options such as surgical repair and medical management, these approaches are often implemented only after the disease has progressed to a certain stage, and they carry inherent risks and limitations.^9^ Therefore, the search for new early intervention and treatment methods is crucial.^10^, ^11^, ^12^ In recent years, advancements in molecular biology and genetic technology have provided some insight into the pathogenesis of AAA, but many areas of uncertainty still require exploration.^13^, ^14^, ^15^

Ferroptosis, as a novel form of cell death, has recently garnered attention in AAA research.^16^, ^17^, ^18^ Ferroptosis relies on the elevation of intracellular iron ions and reactive oxygen species (ROS), leading to lipid peroxidation of cell membranes and, ultimately, cell death.^19^, ^20^, ^21^ Existing studies have indicated the significant role of ferroptosis in atherosclerosis and other vascular pathologies, yet its specific mechanisms in AAA remain unclear.^22^ Particularly, the role of ferroptosis in vascular smooth muscle cells (VSMCs) could be a pivotal aspect in understanding the pathogenesis of AAA.^23^, ^24^, ^25^ VSMCs are a critical cell type for maintaining vascular structure and function, and their damage and death represent a key process in AAA formation.^15^, ^26^ Thus, further exploration of the role of ferroptosis in VSMCs holds significant importance in unraveling the pathogenic mechanisms of AAA.^15^, ^26^

The nuclear factor erythroid 2‐related factor 2 (NRF2)/glutathione peroxidase 4 (GPX4) axis plays a crucial regulatory role in cellular responses to oxidative stress and ferroptosis.^27^, ^28^, ^29^ NRF2, an important transcription factor, induces the expression of various antioxidant genes, reducing cellular oxidative damage.^30^, ^31^, ^32^ GPX4, functioning as a glutathione peroxidase, effectively clears lipid peroxides on cell membranes, thus protecting cells from ferroptotic damage.^33^ Studies have shown that activation of the NRF2/GPX4 axis significantly inhibits ferroptosis, promoting cell survival.^27^, ^29^, ^34^ Therefore, identifying molecules or drugs that can effectively activate the NRF2/GPX4 axis may offer new insights and approaches for the prevention and treatment of AAA.

Extracellular vesicles (EVs) have superior drug encapsulation and delivery capabilities,^35^ as well as better biocompatibility and lower immunogenicity, making them promising next‐generation drug delivery nano‐platforms. For instance, studies show that EVs can facilitate more efficient delivery of RNA and proteins compared to lipid nanoparticles.^36^, ^37^ EVs possess various physiological functions, making them potential natural therapies for a variety of diseases. They inherit functional biomolecules, including proteins, nucleic acids, lipids, and metabolites, from the source cells. Liposomes primarily deliver drugs through passive accumulation in certain tissues unless they carry additional surface ligands. EVs may have inherent targeting capabilities and could potentially deliver functional RNA to other cells^38^ and cross certain biological barriers, such as the blood–brain barrier.^39^ While synthetic drug delivery systems have much lower targeting efficacy than natural drug delivery systems, EVs may provide an efficient natural pathway for transport.^36^ Biomimetic nanovesicles, as a novel drug delivery system, exhibit excellent biocompatibility and targeting properties.^40^, ^41^ The application of nanotechnology enables more precise delivery of drugs to target cells or tissues, thus enhancing therapeutic efficacy and safety.^42^, ^43^ By loading specific mRNA or proteins, biomimetic nanovesicles can efficiently express target genes within specific cells, exerting their therapeutic effects.^44^, ^45^ In this study, we selected phosphatidylethanolamine‐binding protein 1 (PEBP1) mRNA as the target gene and delivered it to VSMCs using biomimetic nanovesicles, aiming to activate the NRF2/GPX4 axis, inhibit ferroptosis, and explore their potential in the prevention and treatment of AAA.

The aim of this study is to utilize biomimetic nanovesicles loaded with PEBP1 mRNA to activate the NRF2/GPX4 axis, inhibit ferroptosis in VSMCs, and prevent the formation of AAA. By validating the efficacy of this novel therapy through in vitro and in vivo experiments, we aim to provide a new strategy for molecular targeted therapy of AAA. Initially, we conducted differential gene expression analysis using AAA‐related transcriptomic datasets downloaded from the GEO database to identify key genes associated with ferroptosis. Subsequently, through single‐cell data analysis, we delved into the molecular characteristics of VSMCs in AAA to confirm the role of PEBP1 in regulating ferroptosis. In vitro experiments were conducted to validate the successful encapsulation and expression of PEBP1 mRNA in nanovesicles and its biological effects in VSMCs. Finally, animal model experiments were carried out to demonstrate the inhibitory effects of nanovesicles on the progression of AAA. This study not only deepens our understanding of the pathogenesis of AAA but also provides potential preventive and therapeutic approaches for clinical application, holding significant scientific research value and clinical prospects.

## 2 MATERIALS AND METHODS

### 2.1 Public data download

We obtained transcriptome data related to AAA from the Gene Expression Omnibus (GEO, https://www.ncbi.nlm.nih.gov/geo/). The dataset GSE57691 consists of 49 samples from AAA patients (GSM1386783–GSM1386831) and 10 samples from normal controls (GSM1386841–GSM1386850).

For single‐cell RNA sequencing (scRNA‐seq) data on AAA, dataset GSE237230 encompasses samples from four AAA patients' abdominal aortas (GSM7597523, GSM7597525, GSM7597527, and GSM7597529). Analysis was conducted using the “Seurat” package in R software. Data quality control was performed based on the criteria: 200 < nFeature_RNA < 5000 and percent.mt < 20, leading to the identification of the top 2000 highly variable genes.

As these data originate from a publicly available database, there is no requirement for ethical approval or informed consent.

### 2.2 Differential expression analysis

Differential expression analysis was performed using the high‐throughput transcriptomic data from the GEO dataset. Genes showing |log2FoldChange| > 1 and *p* < 0.05 were selected as differentially expressed genes (DEGs) with the aid of the R package LIMMA (linear models for microarray data). Subsequently, the “ggplot2” package was utilized to generate volcano plots for visualizing the DEGs. All analyses were carried out in R version 4.3.1 (R Foundation for Statistical Computing).

### 2.3 Selection of ferroptosis‐related genes

A list of known ferroptosis‐related genes was obtained from the FerrDb database (http://www.zhounan.org/ferrdb) and intersected with the DEGs. The “VennDiagram” package (version 1.6.20) in the R environment was used to create Venn diagrams, visually displaying the intersection between the two groups of genes.

### 2.4 scRNA‐seq

In order to reduce the dimensionality of the scRNA‐Seq dataset, principal component analysis (PCA) was performed on the expression profiles of the top 2000 highly variable genes based on variance. The top 20 principal components were selected for downstream analysis using the Elbowplot function in the Seurat software package. Batch correction of the sample data was carried out using the Harmony package, and the principal components (PCs) were ranked by standard deviation using the ElbowPlot. The FindClusters function provided by Seurat was employed to identify main cell subgroups with a default resolution set at res = 1. Subsequently, the uniform manifold approximation and projection (UMAP) algorithm was utilized to reduce the non‐linear dimensionality of the scRNA‐seq sequencing data. Markers for various cell subgroups were identified using the Seurat software package. Annotating the cells was accomplished by leveraging known cell lineage‐specific marker genes and the CellMarker online database in conjunction with the “SingleR” package.

The FindAllMarkers function was employed to analyze the characteristic genes in each cell type after clustering, with a threshold of *p* < 0.05 for selection criteria, resulting in the identification of characteristic genes for VSMCs.

### 2.5 Cell extraction and cultivation

Primary VSMCs were isolated from the aorta of AAA model mice (for subsequent animal modeling) through the following process. Mice were euthanized with isoflurane inhalation, and the entire aortic section was dissected. The outer membrane was carefully removed after a continuous 30‐min digestion at 37°C with 347 U/mg of Type II collagenase (C2‐BIOC, Sigma‐Aldrich, USA). The endothelium was removed by gently rubbing with a sterile cotton‐tipped applicator, and the aorta was diced into small pieces. Subsequently, a mixture of 347 U/mg collagenase II and 6 U/mg elastase IV (E0258, Sigma‐Aldrich, USA) was used for a 30‐min digestion at 37°C. The cells were then cultured in Dulbecco's Modified Eagle Medium (DMEM, 11965092, Gibco, USA) supplemented with 10% fetal bovine serum (FBS, A5670701, Gibco, USA), 1% smooth muscle growth supplement (S00725, Gibco, USA), and 1% penicillin–streptomycin (15140148, Gibco, USA). Identification of the cells was conducted using α‐Smooth Muscle Actin (α‐SMA) staining, a specific marker for VSMCs, revealing that over 98% of the cells were α‐SMA positive.^46^

### 2.6 Cell lentiviral transfection

Lentivirus infection: The packaging virus and the target vector were co‐transfected into Human Embryonic Kidney 293T cells (HEK293T, CBP60661, Nanjing KeyGen Biotech Co., Ltd., Jiangsu, China) using Lipofectamine 2000 (11668500, Invitrogen™). After 48 h of cell culture, the supernatant was collected, which contained viral particles after filtration and centrifugation. The virus was collected during the exponential growth phase, and the viral titer was determined. Lentivirus overexpressing PEBP1 was constructed and packaged by GeCai Gene, with the lentivirus gene overexpression vector being LV‐PDGFRA. During the logarithmic growth phase of VSMCs, they were digested using trypsin, dissociated, and prepared into a cell suspension at a concentration of 5 × 10^4^ cells/mL. Subsequently, the cells were seeded into a six‐well plate, with each well containing 2 mL of cell suspension. Before grouping, the lentiviral transfection titers and times were optimized. Pre‐experiment infection was performed with lentivirus at three different MOIs: 1 × 10^8^, 1 × 10^7^, and 1 × 10^6^ TU/mL, incubating for 24, 48, or 72 h. After selecting the best transfection conditions, stable cell lines were selected using 2 μg/mL puromycin (HY‐K1057, Med Chem Express, USA) for 2 weeks.^47^

Cell grouping: The cells were divided into four groups: oe‐NC group (infected with lentivirus overexpressing empty vector), oe‐PEBP1 group (infected with lentivirus overexpressing PEBP1), PBS group (treated with phosphate‐buffered saline), EVs group (untreated mRNA‐loaded EVs for 48 h), and PEBP1‐EVs group (treated with 20 μg of PEBP1‐EVs for 48 h).^48^

### 2.7 Cell immunofluorescence

Cells were rinsed with cold PBS and fixed with 4% paraformaldehyde (PFA) (P885233, Macklin, Shanghai, China) for 15–30 min. Subsequently, the cells were treated with 0.1% Triton (L885651, Macklin, Shanghai, China) for 15 min to permeabilize the cell membrane. After two PBS washes, the cells were cultured in PBS containing 15% FBS at room temperature for 1 h. The cells were then incubated overnight at 4°C with rabbit monoclonal antibody anti‐α‐SMA (ab150301, 1 μg/mL, Abcam, UK), followed by a 1‐h incubation with goat anti‐rabbit IgG H&L (FITC) (ab6717, 1:200, Abcam, UK). After three PBS washes, the cells were incubated with DAPI (ab104139, 1:1000, Abcam, UK) for 10 min, followed by three PBS washes. Cell immunofluorescence was observed using a fluorescence microscope (Zeiss Observer Z1, Germany). Five randomly selected fields were chosen to calculate the ratio of α‐SMA‐positive cells per field, and the mean value was determined.

### 2.8 Quantitative reverse transcription polymerase chain reaction analysis

Total RNA was extracted from cells in each group using the Trizol reagent kit (T9424, Sigma‐Aldrich). The quality and concentration of RNA were determined using a UV–visible spectrophotometer (ND‐1000, Nanodrop, USA). For mRNA expression analysis, reverse transcription was performed using the PrimeScript™ RT kit (RR014B, TaKaRa, Japan). Real‐time RT‐qPCR was carried out on an ABI 7500 PCR system (Applied Biosystems, USA) using the TB Green® Premix Ex TaqTM kit (RR420W, TaKaRa, Japan) with the sequences detailed in Table 1. GAPDH was used as an internal reference for mRNA expression. Relative quantification was performed by comparing Ct values, with the formula 2^−ΔΔCt^ representing the fold change in expression of the target gene between the experimental and control groups, where ΔΔCT = ΔCt experimental group − ΔCt control group, and ΔCt = Ct target gene − Ct internal reference gene.

**TABLE 1:** RT‐qPCR primer sequences.

Gene name	Primer sequences
PEBP1 (mouse)	F: 5′‐TCCCATCTTAGCTGAGCCCT‐3′
	R: 5′‐AGGATCAACACACTCGGCAG‐3′
NRF2 (mouse)	F: 5′‐GCCCTCAGCATGATGGACTT‐3′
	R: 5′‐AACTTGTACCGCCTCGTCTG‐3′
GPX4 (mouse)	F: 5′‐GCCAAAGTCCTAGGAAACGC‐3′
	R: 5′‐CCGGGTTGAAAGGTTCAGGA‐3′
GAPDH (mouse)	F: 5′‐CCCTTAAGAGGGATGCTGCC‐3′
	R: 5′‐TACGGCCAAATCCGTTCACA‐3′

### 2.9 Western blot analysis

Cellular or tissue total protein was extracted using efficient RIPA lysis buffer (R0010, Solarbio, Beijing, China) following the manufacturer's instructions. After 15 min of lysis at 4°C, the lysates were centrifuged at 12,000 × *g* for 15 min, and the supernatant was collected for protein concentration determination using the BCA assay kit (20201ES76, Yisheng Biotechnology Co., Ltd., Shanghai, China) for each sample. Following quantification based on different concentrations, the proteins were separated by polyacrylamide gel electrophoresis, transferred to a PVDF membrane using a wet transfer system, and blocked with 5% BSA at room temperature for 1 h. Primary antibodies including PEBP1 (ab76582, 1:1000, Abcam, UK), NRF2 (80593‐1‐RR, Proteintech), GPX4 (ab125066, 1:1000, Abcam, UK), Calnexin (ab265602, 1:10000, Abcam, UK), CD63 (ab134045, 1:5000, Abcam, UK), CD8 (ab79559, 1:500, Abcam, UK), and GAPDH (ab9485, 1:1000, Abcam, UK) were then incubated overnight at 4°C, followed by washing the membrane with TBST for 5 min ×3 times. Subsequently, the membrane was incubated with an HRP‐conjugated goat anti‐rabbit IgG (ab205718, 1:20,000, Abcam, UK) dilution at room temperature for 1 h. After another round of washing with TBST for 5 min ×3 times, the membrane was developed using an ECL kit. Quantitative protein analysis was performed using ImageJ software (v1.48, National Institutes of Health, USA) by quantifying the protein levels based on the ratio of the grayscale intensity of each protein to that of the internal control GAPDH. The baseline groups for the in vitro experiments were the oe‐NC (overexpression negative control) group and the PBS and blank EVs groups for in vivo experiments.

### 2.10 Cell counting kit‐8 assay

VSMCs were subjected to experiments using the cell counting kit‐8 (CCK‐8) (Beyotime, C0037, Shanghai, China). The cells were seeded in a 96‐well plate and treated, with 10 μL of CCK‐8 solution added to each well at 24, 48, and 72 h. Subsequently, the cells were further incubated for 1 h, and the absorbance of the cells at a wavelength of 450 nm was measured using the BioTek ELx808 microplate reader (Biotek, USA). Each group was performed with six replicates, and the experiment was repeated three times for reliability.

### 2.11 Flow cytometry analysis

VSMCs were seeded at a density of 1 × 10^5^ cells per well and washed in chilled PBS. Subsequently, they were stained in the dark for 15 min using a detection kit (APOAF‐20TST, Sigma‐Aldrich, USA). Following this, the pellet was resuspended in 400 μL of binding buffer and stained with 5 μL of Annexin‐V provided in the kit. Flow cytometry was employed to analyze the cells. Cells in the upper right quadrant showing Annexin V + PI + phenotype indicated late apoptotic cells; cells in the lower right quadrant with Annexin V + PI − phenotype represented early apoptotic cells; cells in the upper left quadrant exhibiting Annexin V − PI + phenotype denoted necrotic cells; and cells in the lower left quadrant displaying Annexin V − PI − phenotype signified live cells.

### 2.12 Measurement of ROS levels

The level of ROS in VSMCs was determined using the DCFH‐DA fluorescence staining method. VSMCs (5 × 10^4^ cells) were stained with 10 μM DCFH‐DA (S0033S, Shanghai Biyun Tian Biotechnology Co., Ltd.) at 37°C for 30 min, followed by observation under a fluorescence microscope (Nikon, TE2000‐S, Japan). The fluorescence intensity was measured using Carl Zeiss AxioVision 4.8 software, enabling the detection of intracellular ROS production.

### 2.13 Determination of Fe^2+^ content

The supernatant from cell culture medium in each group was collected, and the iron levels in the cells were measured using an iron assay kit (ab83366, Abcam, UK) following the manufacturer's instructions.

### 2.14 Observation of mitochondrial damage using transmission electron microscopy

Mitochondrial damage in VSMCs was observed using transmission electron microscopy (TEM). Samples were fixed overnight in a 2.5% glutaraldehyde solution at 4°C, followed by fixation in a 1% osmium tetroxide solution for 1–2 h at room temperature. Dehydration was carried out with graded ethanol (50%, 70%, 80%, 90%, and 95%), followed by treatment with pure acetone and overnight embedding in pure embedding resin. The samples were then embedded after undergoing permeation treatment. Subsequently, the embedded samples were heated at 70°C overnight to obtain properly embedded samples. Thin sections of the samples were cut using the UM10 ultramicrotome (Jiangsu Leibo Scientific Instrument Co., Ltd., China), stained with lead citrate solution and 50% ethanol‐saturated uranyl acetate for 15 min each, enabling observation under the transmission electron microscope. Mitochondrial status was assessed by TEM, based on membrane integrity and cristae structure.

### 2.15 Enrichment of EVs

VSMCs were harvested and subjected to conditioned culture. The cells were centrifuged at 300 × *g* for 5 min at room temperature to remove detached cells. The supernatant was then transferred to 4°C and centrifuged at 2000 × *g* for 10 min to eliminate dead cells. The obtained supernatant was further centrifuged at 10,000 × *g* for 30 min at 4°C to remove cell debris. Subsequently, the supernatant was transferred to ultracentrifuge tubes and centrifuged at 100,000 × *g* for 70 min at 4°C to obtain a pellet. The pellet was washed with PBS (P1020, Solarbio, Beijing, China) and centrifuged again at 100,000 × *g* for 70 min under the same conditions to obtain the final pellet. Finally, the pellet was resuspended in PBS and stored at −80°C. All ultracentrifugation steps were performed using the Beckman Optima MAX‐XP ultracentrifuge, while other centrifugation steps were carried out using the Beckman Allegra X‐15R desktop centrifuge.

### 2.16 mRNA‐loaded EVs

PEBP1 mRNA designed by Trilink company was synthesized and obtained. Initially, purified mRNA was prepared for loading, followed by the addition of lipid‐coated mRNA into the EVs through a mixing and incubation process. Subsequently, the Gene Pulser Xcell™ electroporation system (Bio‐Rad, USA) was utilized with settings at 200 V and 950 μF for electroporation. After electroporation, the samples were left to recover at room temperature for 30 min to facilitate mRNA encapsulation.

### 2.17 mRNA encapsulation efficiency detection

The encapsulation efficiency was determined using RiboGreen RNA (R11490; Thermo Fisher Scientific, USA). Briefly, the sample was incubated with RiboGreen reagent in 1 × TE buffer (12090015, Invitrogen, USA), with or without 0.5% Triton X‐100 (T109027, Aladdin®). The total mRNA and unencapsulated mRNA were measured. Fluorescence intensity was measured at an excitation wavelength of 485 nm and an emission wavelength of 528 nm. The encapsulation efficiency was calculated using the formula: EE% = ((total mRNA − unencapsulated mRNA)/total mRNA) × 100%.

For all groups, the mRNA encapsulation efficiency was greater than 85%.^49^, ^50^

### 2.18 Characterization of nanovesicles

Nanoparticle tracking analysis (NTA): EVs were diluted in PBS at a ratio of 1:100. The prepared EV samples were placed in the Nanosight LM10‐HS system (Malvern Panalytical). For each EV sample, the system recorded a 30‐s video to capture the movement trajectories of EV particles in PBS, repeating this process three times.

TEM: 10 μL of PEBP1‐EVs suspension was fixed in 4% PFA and then air‐dried onto electron microscopy grids coated with formvar carbon. Subsequently, staining was performed with 3% phosphotungstic acid (G1871, Soleibao) for 5 min. Finally, EV morphology was thoroughly observed and analyzed using the JEOL 1010 transmission electron microscope.

Surface marker identification was performed using WB analysis. The protein content was determined and standardized using the BCA protein assay kit (23227, Thermo Fisher, USA). A protein concentration of 50 μg/mL was used in the experiment. SDS–PAGE gels were prepared for protein denaturation and electrophoresis. Subsequently, transfer was carried out, and the expression of EVs marker proteins CD63 (ab315108, Abcam, UK) and CD81 (ab109201, Abcam, UK) was detected, along with the common contaminant endoplasmic reticulum protein Calnexin (ab22595, Abcam, UK) as a negative control to ensure extraction purity.

### 2.19 Stability detection of mRNA‐loaded nanovesicles

We stored mRNA‐loaded biomimetic nanovesicles at 4°C for 28 days. The stability was monitored every 7 days using particle size and Zeta potential measurements.^51^

### 2.20 Uptake of nanovesicles

Purified EVs were labeled with PKH67 green fluorescent dye (D0031, Solarbio, Beijing, China). The PKH67 dye was diluted in Diluent C (10‐fold dilution) to prepare a fresh dye solution. Subsequently, the PEBP1‐EVs suspension was mixed with the dye solution at room temperature and incubated for 10 min, followed by the addition of 2 mL of PBS containing 1% FBS to terminate the staining process. The labeled EVs were co‐incubated with VSMCs/HEK293T (3 × 10^5^ cells) for 12 h, then washed three times with PBS to remove any uninternalized outer vesicles. The cells were fixed using 4% PFA (P1110, Solarbio, Beijing, China), washed twice with PBS, and stained with DAPI (D9542, 1:1000, Sigma‐Aldrich, USA) for nuclear visualization. Finally, the uptake of EVs by VSMCs was observed using a fluorescence microscope (Olympus IX73, Japan).

### 2.21 Establishment of an AAA mouse model

All animal experiments in this study were approved by the Ethics Committee of the First Affiliated Hospital of Wenzhou Medical University. All procedures were conducted in strict accordance with the “Guidelines for the Care and Use of Laboratory Animals” and the ARRIVE guidelines (https://arriveguidelines.org/arrive-guidelines) to ensure compliance with ethical and welfare standards. Animals were randomly assigned to experimental groups using a computer‐generated randomization method to minimize bias. Given the potential protective effects of estrogen, as reported in previous studies,^47^, ^52^ only male mice were used in this study. The impact of using a single sex on the generalizability and clinical translation of the findings is acknowledged, and future research will incorporate both male and female animals to assess potential sex‐based differences.

Twenty‐four male C57BL/6J mice aged 5–6 months were purchased from Beijing Vital River Laboratory Animal Technology Co., Ltd. The mice were housed in IVC cages under specific pathogen‐free (SPF) conditions in a facility maintained at a temperature of 22–26°C and a relative humidity of 45%–60%. Standard food and water were provided ad libitum. The light–dark cycle was set to 12 h each.

The mice were initially randomly divided into four groups: Sham, AAA, AAA + EVs, and AAA + PEBP1‐EVs. The Sham group received 0.9% NaCl, while the AAA group received CaCl_2_ induction. The AAA modeling procedure involved anesthetizing the mice, performing laparotomy, and isolating the structures below the renal artery and above the arterial bifurcation of the abdominal aorta. Subsequently, a cotton cloth containing 0.5 mol/L CaCl_2_ was placed on the outer surface of the aorta for 20 min, followed by rinsing with sterile 0.9% saline and closure of the incision. Four weeks after CaCl_2_ stimulation, all mice in the modeling groups were euthanized accordingly. PEBP1‐EVs and AAA + EVs treatment were applied to the AAA + PEBP1‐EVs group post‐modeling, with a weekly injection of 2 × 10^10^ PEBP1‐EVs or EVs (approximately 100 μg protein) per mouse for a duration of 4 weeks. After euthanizing the mice, 0.9% saline was slowly and evenly injected into the left ventricle at a controlled rate, while the right atrial appendage was incised to allow blood drainage until the blood color was no longer visible, after which the PBS injection was stopped. The aorta (from the aortic arch to the iliac bifurcation) was excised, placed in a dish filled with 0.9% saline, dissected under a dissecting microscope to remove fat and connective tissues adhering to the outer aortic membrane, and photographs of the aorta were taken.^53^, ^54^

### 2.22 Evaluation of maximum abdominal aortic diameter by echocardiography

Echocardiographic assessments were conducted using the Vevo 2100 console (Visual Sonics Vevo 2100, FUJIFILM, Bothell, WA, USA) to analyze the vascular diameter at 28 days post‐modeling. B‐mode ultrasound data of the diastolic aorta were obtained with a transducer (MS550D) operating at a central frequency of 40 MHz and a focal length of 7 mm for imaging the murine abdominal aorta. Analysis was performed using ultrasound statistical software to measure the maximum dilated segment of the aorta. Arterial aneurysm formation was defined as an increase of at least 50% in the outer width of the suprarenal aorta.

### 2.23 Hematoxylin and eosin staining

The abdominal aortas of each group of mice were collected, exposed, and photographed after removing surrounding tissues under a dissecting microscope. Subsequently, the abdominal aortas were fixed in 4% PFA (P0099, Shanghai Biyun Tian Biotechnology Co., Ltd). The aortas were sectioned into 7 μm thick slices for further analysis. The prepared sections were stained in hematoxylin solution (H8070, Solarbio, Beijing, China) for 5–10 min at room temperature. After rinsing the slides with distilled water, they were dehydrated in 95% ethanol, immersed in eosin solution (G1100, Solarbio, Beijing, China) for 5–10 min, and underwent routine dehydration, clearing, and mounting for microscopic observation.

### 2.24 Tissue immunofluorescence detection of EVs homing location

To investigate the internalization of biomimetic nanovesicles in the aortic wall of mice, 5^×9^ DiI red fluorescent dye (C1036, Beyotime) was injected intravenously to label PEBP1‐EVs (DiI‐PEBP1‐EVs). After 24 h, tissues were collected and stored at −80°C. Aortic tissues from at least six mice per group were sectioned into 5 μm cross‐sections and incubated overnight at 4°C with the primary antibody PEBP1 (ab76582, 1:100, Abcam, UK). The sections were then incubated for 1 h at room temperature with goat anti‐rabbit secondary antibody (Alexa Fluor® 488) (ab150077, 1:500, Abcam, UK) and stained with DAPI (ab104139, 1:1000, Abcam, UK) for nuclear labeling. Images were captured using a fluorescence microscope (TI‐S, Nikon). Six random areas from each tissue were photographed, and the average value was used in subsequent statistical analysis.^47^

### 2.25 ELISA

Mouse sera were collected, and levels of C‐reactive protein (CRP) and matrix metalloproteinase 2 (MMP2) were measured using Mouse ELISA kits for CRP (ab222511, Abcam, UK) and MMP2 (ab254516, Abcam, UK) following the manufacturer's instructions.

### 2.26 Statistical analysis

The data, obtained from at least three independent experiments, are presented as mean ± standard deviation (mean ± SD). Two‐group comparisons were made using an independent two‐sample *t*‐test, with sample size determined based on *t*‐test calculations using SPSS and the appropriate formula. An one‐way analysis of variance (ANOVA) was employed for comparisons involving three or more groups. In cases where ANOVA indicated significant differences, post hoc Tukey's Honestly significant difference (HSD) test was conducted to compare differences between groups. For non‐normally distributed or unequal variance data, the Mann–Whitney *U* test or Kruskal–Wallis *H* test was applied. Statistical analyses were performed using GraphPad Prism 9 (GraphPad Software, Inc.) and R programming language. The significance level for all tests was set at 0.05, with a two‐sided *p*‐value less than 0.05 considered statistically significant.

## 3 RESULTS

### 3.1 Transcriptomic analysis reveals the key role of ferroptosis genes in AAA development

**FIGURE 1:**
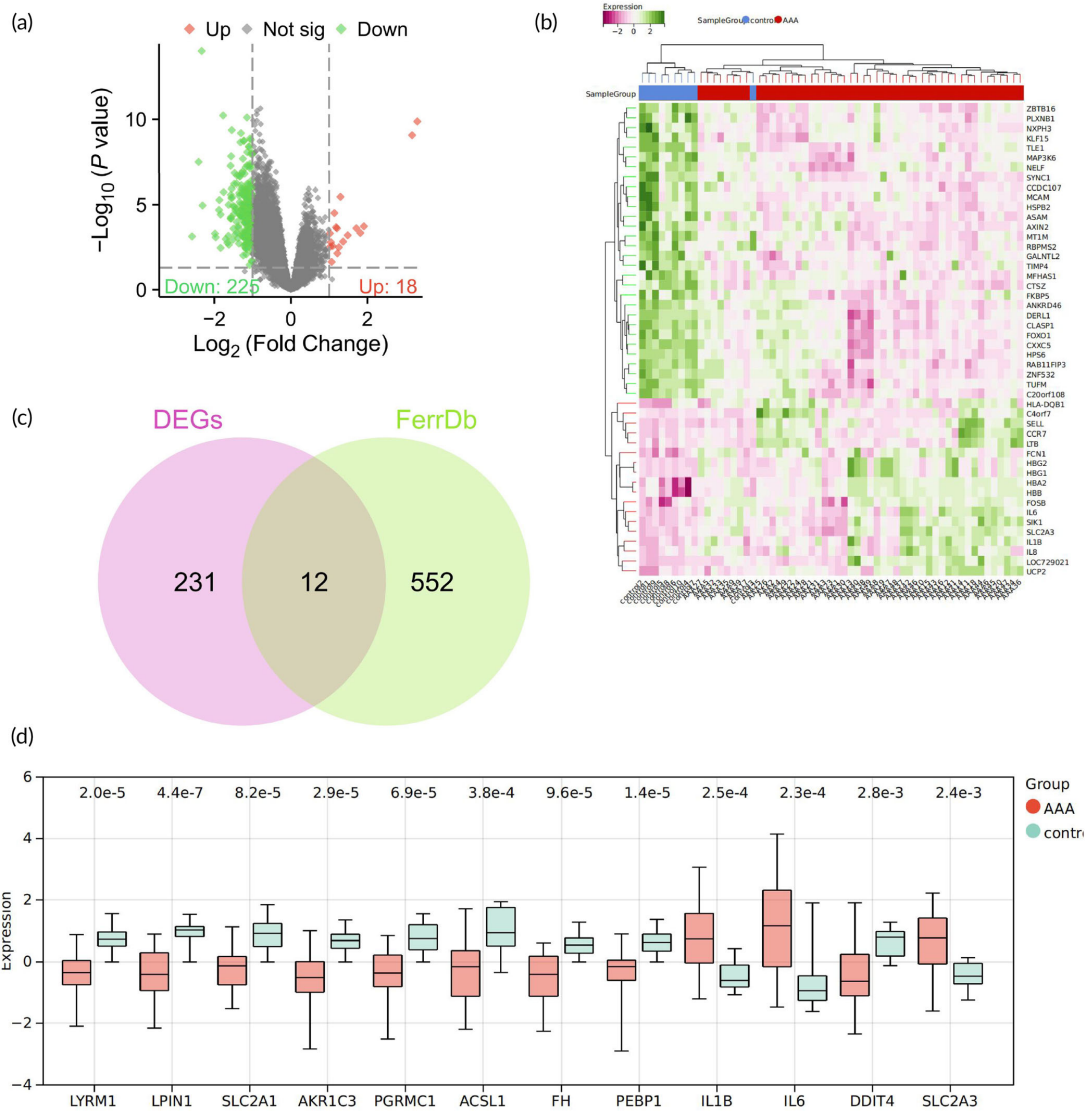
Expression and regulation analysis of ferroptosis genes in the development of AAA. (a) Differential analysis volcano plot of AAA samples compared to normal samples, with red indicating significantly upregulated genes, green indicating significantly downregulated genes, and gray indicating genes with no significant difference. (b) Heatmap of the top 30 DEGs. (c) Venn diagram showing the intersection between DEGs and ferroptosis‐related genes in the FerrDb database. (d) Boxplot displaying the expression of 12 intersecting genes in the AAA dataset. AAA: *N* = 49; control: *N* = 10.

AAA is a localized dilation of the abdominal aorta,^55^ posing a significant public health concern, yet the medical needs for treatment remain unmet.^56^ Initially, the AAA‐related transcriptomic dataset GSE57691 was downloaded from the GEO database. Differential analysis was conducted using the LIMMA package, identifying 243 significantly DEGs, comprising 225 significantly downregulated genes and 18 significantly upregulated genes (Figure 1a,b).

Ferroptosis is a programmed form of cell death characterized by iron‐dependent lipid peroxidation.^15^ Previous studies have indicated that preventing ferroptosis can help in the prevention of AAA.^26^ A total of 564 ferroptosis‐related genes were obtained from the FerrDb database, and 12 genes were found to intersect with the aforementioned DEGs (Figure 1c). The expression profiles of these 12 intersecting genes in the AAA transcriptomic dataset revealed significant upregulation of IL1B, IL6, and SLC2A3 in the AAA group, while the remaining nine genes showed significant downregulation (Figure 1d).

By comparing the ferroptosis‐related genes in the FerrDb database with the DEGs, 12 intersecting genes were identified. The expression patterns of these genes in the AAA group reveal the potential role of ferroptosis‐related genes in the development of AAA.

### 3.2 Single‐cell analysis reveals the key role of PEBP1 in ferroptosis‐mediated cell death in AAA

In the aforementioned transcriptional chip analysis of AAA, we identified 12 ferroptosis‐related genes that regulate AAA. To gain a deeper understanding of the cellular heterogeneity in AAA and identify crucial ferroptosis‐related genes, we acquired the AAA single‐cell transcriptome chip GSE237230 from the GEO database. Following data integration using the Seurat package, we filtered out high‐quality cells based on criteria such as nFeature_RNA < 5000, nCount_RNA < 20,000, and percent.mt < 20%. This filtering yielded an expression matrix containing 16,511 genes and 3233 cells (Figure S1). The sequencing depth correlation analysis revealed a correlation coefficient of *r* = −0.15 between nCount_RNA and percent.mt, and a correlation coefficient of *r* = 0.85 between nCount_RNA and nFeature_RNA in the filtered data (Figure S1), indicating that the quality of the filtered cell data was suitable for subsequent analysis.

Following this, we further analyzed the filtered cells and initially normalized the data. Subsequently, based on the selected highly variable genes, we performed linear dimensionality reduction using PCA (Figure S1), illustrating the distribution of cells in PC1 and PC2 (Figure S1). The results indicated the presence of batch effects among the samples.

We conducted batch correction on the sample data using the Harmony package and performed standard deviation‐based ordering of PCs through an Elbow Plot (Figure S1). The results post‐correction indicated a substantial elimination of batch effects (Figure S2). Next, we utilized the UMAP algorithm for nonlinear dimensionality reduction on the top 20 PCs and showcased the clustering patterns at various resolutions using the cluster package (Figure S2). Through UMAP clustering analysis, we categorized all cells into 18 cell clusters (Figure S2).

**FIGURE 2:**
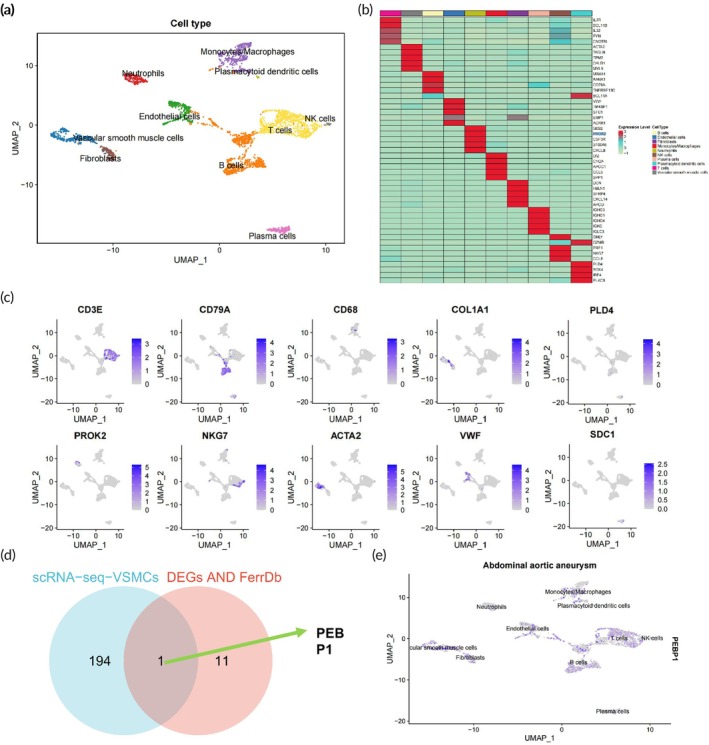
Single‐cell expression characterization of ferroptosis‐related genes in AAA. (a) Visualization of cell annotation results grouped based on UMAP clustering. (b) Correlation heatmap of the top five genes ranked by expression in 10 cell types. (c) UMAP plot of 10 Marker genes. (d) Venn diagram displaying the intersection between VSMCs' feature genes and 12 ferroptosis‐related DEGs. (e) UMAP plot of PEBP1. AAA: *N* = 4.

Subsequently, we utilized the Bioconductor/R software package “SingleR” in conjunction with manual annotation to automatically annotate the 18 cell clusters, resulting in the identification of 10 cell types: Neutrophils, T cells, NK cells, monocytes/macrophages, B cells, VSMCs, plasmacytoid dendritic cells, fibroblasts, endothelial cells, and plasma cells (Figure 2a). Furthermore, a heatmap displaying the top five expressed genes within these 10 cell types indicated the reliability of cell clustering (Figure 2b). The UMAP plot further illustrated the distribution of marker genes for each cell type (Figure 2c).

In AAA, VSMCs progressively diminish, and the extracellular matrix (ECM) sustains damage, playing a crucial role in AAA development.^57^ In this context, we utilized the FindAllMarkers function to identify differential characteristic genes of VSMCs, resulting in 195 genes. Intersecting these with the previously identified 12 ferroptosis‐related DEGs, we identified a unique intersection gene, PEBP1 (Figure 2d). PEBP1 exhibits widespread expression across various cell types (Figure 2e). Studies have indicated the capability of PEBP1 to suppress ferroptosis,^58^ and existing reports confirm a significant downregulation of PEBP1 expression in AAA.^59^

This study, through single‐cell transcriptome data analysis, unveils the expression characteristics of ferroptosis‐related genes in AAA. PEBP1, as the sole intersecting gene, shows significantly reduced expression in AAA and is known to possess ferroptosis‐inhibiting properties, suggesting its potentially pivotal role in the pathogenesis of AAA.

### 3.3 PEBP1 reduces ferroptosis and apoptosis in VSMCs by inhibiting ROS and iron ion levels

**FIGURE 3:**
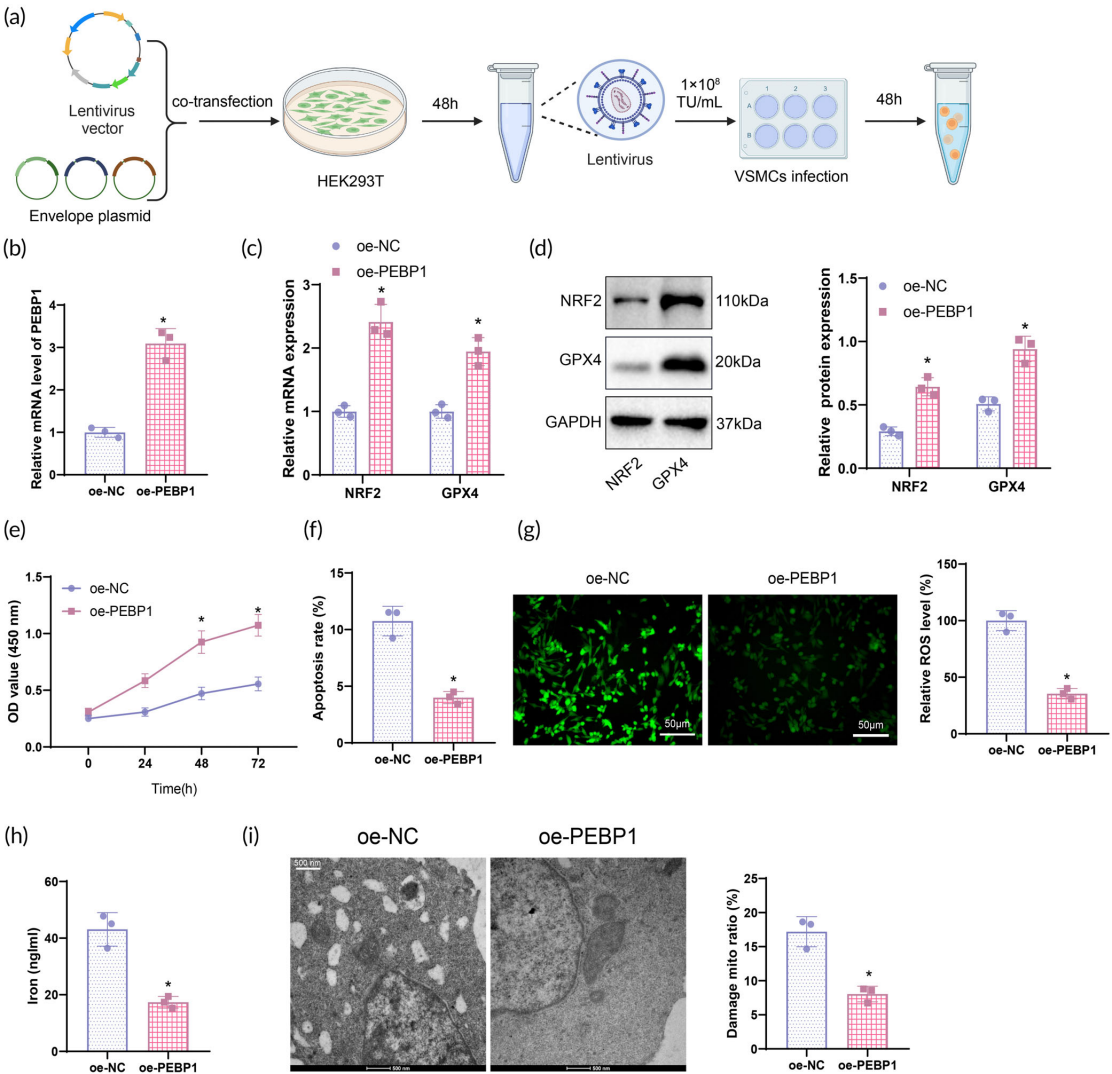
PEBP1 modulates ferroptosis and proliferation apoptosis in VSMCs by inhibiting ROS and iron levels. (a) Diagram of the specific procedure for overexpression and measurement time points. (b) RT‐qPCR analysis of PEBP1 mRNA expression in VSMCs of each group. (c) RT‐qPCR analysis of NRF2 and GPX4 mRNA expression in VSMCs of each group. (d) WB detection of NRF2 and GPX4 protein expression in VSMCs of each group. (e) CCK‐8 assay measuring VSMCs' proliferation capacity. (f) Flow cytometry assessment of VSMCs' apoptosis rate. (g) DCFDA fluorescence probe analysis of ROS levels in VSMCs (scale bar: 50 μm). (h) Detection of iron ion levels in VSMCs of each group using a kit. (i) TEM observation of the cellular structural features of VSMCs in each group (scale bar: 500 nm). *Compared to the oe‐NC group, *p* < 0.05. Statistical comparisons between the two groups were conducted using a *t*‐test, while cell viability at different time points was analyzed using a two‐way ANOVA. Cell experiments were repeated three times.

Based on the aforementioned bioinformatics analysis, we identified gene expression characteristics related to ferroptosis in AAA. PEBP1 was the only gene in the intersection, showing significantly low expression in AAA. Known for its role in inhibiting ferroptosis, PEBP1's reduced expression in AAA implies its potential crucial involvement in the pathogenesis of this disease. Primary VSMCs were isolated from mouse aortas, and immunofluorescence staining of α‐SMA (a VSMC‐specific marker) revealed that over 98% of cells were α‐SMA positive (Figure S3). To investigate the impact of PEBP1 expression in VSMCs on the expression of ferroptosis‐related factors NRF2 and GPX4 (Figure 3a), we first performed PEBP1 overexpression. The optimal conditions for lentiviral infection were determined to be 1 × 10^8^ TU/ml for 48 h (Figure S3), and these conditions were used for verification. RT‐qPCR results confirmed successful overexpression of PEBP1 (Figure 3b). Subsequently, RT‐qPCR and WB analyses of NRF2 and GPX4 expression in VSMCs from different groups showed a significant increase in NRF2 and GPX4 expression in the oe‐PEBP1 group compared to the oe‐NC group (Figure 3c,d).

The CCK‐8 assay revealed that the proliferation capacity of VSMCs in the oe‐PEBP1 group was significantly increased compared to the oe‐NC group (Figure 3e), while the apoptosis rate was significantly reduced (Figure 3f). Flow cytometry was utilized to detect the apoptosis rate in VSMCs. ROS and iron ion levels in VSMCs from different groups were assessed using a fluorescence probe, DCFDA, and the respective assay kits. The results showed a significant decrease in ROS and iron ion levels in VSMCs of the oe‐PEBP1 group compared to the oe‐NC group (Figure 3g,h). TEM observation demonstrated that VSMCs in the oe‐PEBP1 group had more intact mitochondrial structures and fewer instances of cell membrane rupture compared to the oe‐NC group (Figure 3i). Activation of PEBP1 effectively inhibits the accumulation of ROS and iron ions during ferroptosis, thus protecting cells from ferroptosis‐induced damage. This finding provides crucial molecular support for potential therapeutic strategies in the treatment of AAA.

### 3.4 Successful mRNA‐loaded nanovesicles carrying PEBP1 mRNA

EVs are lipid membrane‐bound vesicles secreted by cells, carrying biological substances such as proteins, lipids, and nucleic acids. In recent years, due to their ability to promote wound healing and tissue regeneration, EVs have garnered significant attention.^60^ mRNA can be delivered to cells in vivo and in vitro through EVs, with exosomes considered to be normal biological nanovesicles.^61^ Consequently, we have successfully prepared nanovesicles carrying PEBP1 mRNA.

**FIGURE 4:**
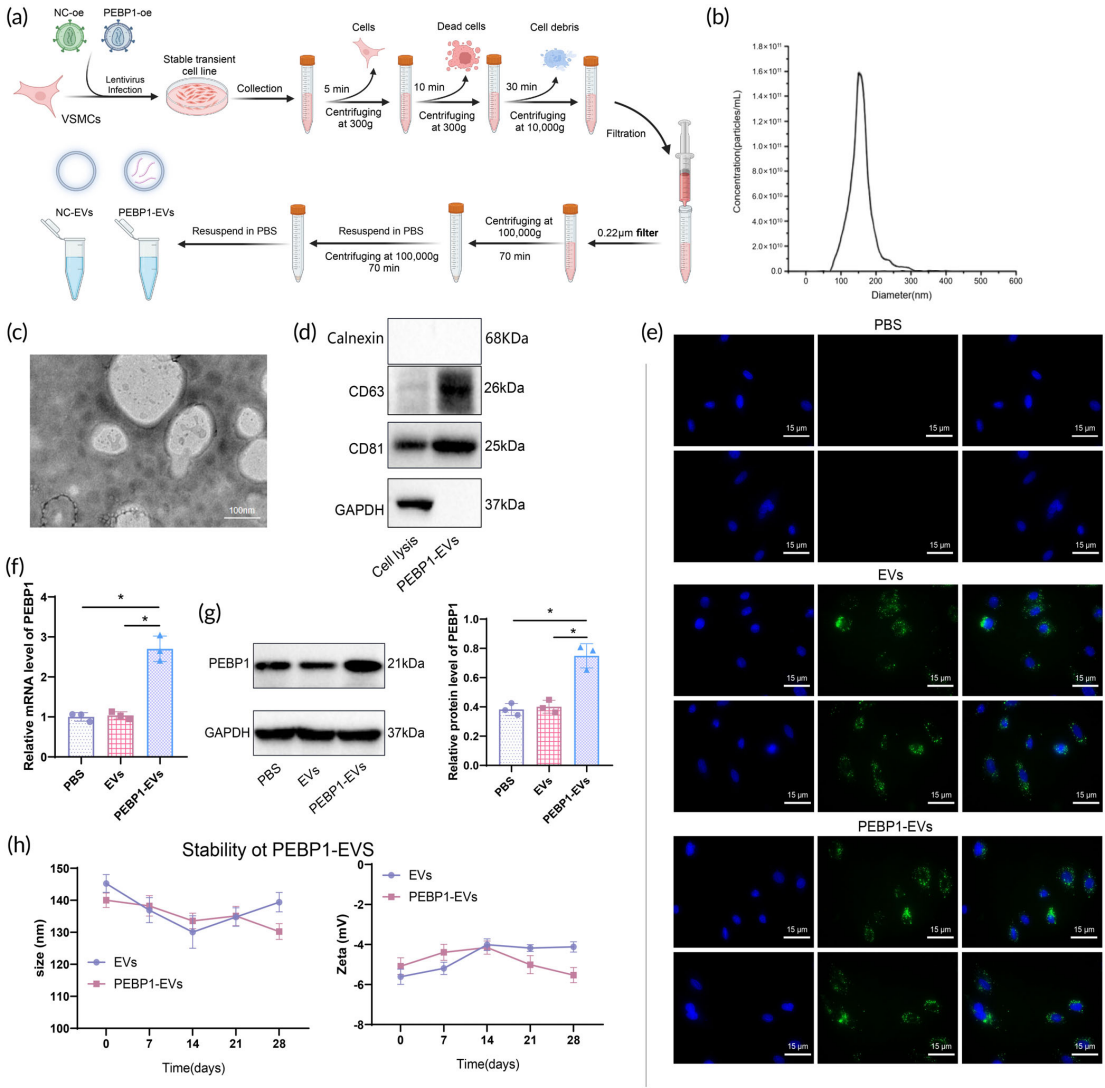
Preparation of biomimetic nanovesicles loaded with PEBP1 mRNA. (a) EVs separation process. (b) NTA analysis revealing the average size of nanovesicles. (c) TEM observation of nanovesicle structure (scale bar: 100 nm). (d) Expression of exosomal markers CD63 and CD81, and endoplasmic reticulum protein Calnexin detected by WB in nanovesicles. (e) Fluorescence confocal microscopy showing cellular uptake of nanovesicle structures by VSMCs (scale bar: 15 μm). (f) RT‐qPCR evaluating the levels of PEBP1 mRNA in VSMCs post‐treatment with biomimetic nanovesicles loaded with PEBP1 mRNA. (g) WB analyzing PEBP1 protein levels in VSMCs post‐treatment with biomimetic nanovesicles loaded with PEBP1 mRNA. (h) Stability testing of mRNA‐loaded nanovesicles. *Significance compared to the PBS group (*p* < 0.05). Multiple comparisons were conducted using one‐way ANOVA, while different time points' cell viability was analyzed using two‐way ANOVA. Cell experiments were performed in triplicate.

We cultured VSMCs, induced their secretion of exosomes, and successfully achieved electroporation of PEBP1 mRNA, leading to the preparation of biomimetic nanovesicles (Figure 4a). NTA revealed that the average diameter of these nanovesicles was 120 ± 50 nm, demonstrating good uniformity and a suitable size for biomedical applications (Figure 4b). TEM observations confirmed that these nanovesicles exhibited regular spherical structures and distinct membrane features (Figure 4c). Furthermore, WB results indicated the surface expression of typical exosome markers CD63 and CD81 on these nanovesicles without the expression of endoplasmic reticulum protein Calnexin, further establishing their exosome characteristics (Figure 4d).

Following the treatment of VSMCs/HEK293T with biomimetic nanovesicles loaded with PEBP1‐EVs, the cellular uptake and impact on PEBP1 gene expression in different cells were evaluated. First, we demonstrated that EVs derived from VSMCs were preferentially taken up by VSMCs compared to HEK293T cells (Figure S4). RT‐qPCR and WB analysis also showed higher PEBP1 levels in VSMCs (Figure S4). After confirming the preferential uptake by VSMCs, we treated VSMCs with different doses of PEBP1‐EVs to investigate the relationship between EV dose and PEBP1 levels in the receptor VSMCs. The results showed that PEBP1 expression increased with the dosage (Figure S4). Considering the negative effects of excessively high doses on EVs and cells, we selected 20 μg for subsequent experiments. Fluorescence confocal microscopy observations revealed significant internalization of fluorescently labeled nanovesicles within cells after 24 h of treatment (Figure 4e). RT‐qPCR and WB analyses confirmed a substantial increase in PEBP1 mRNA and protein expression levels in VSMCs post‐treatment, indicating the successful delivery of PEBP1 mRNA into the cells by the nanovesicles (Figure 4f,g). Additionally, we assessed the stability of mRNA‐loaded biomimetic nanovesicles stored at 4°C for 28 days. Particle size and Zeta potential were measured every 7 days. The results showed that the average diameter of the nanovesicles remained stable at 120 ± 50 nm, and the Zeta potential remained stable over time (Figure 4h).

These findings demonstrate the successful preparation of nanovesicles capable of efficiently delivering genetic material and achieving gene expression in target cells, laying the experimental groundwork for future therapeutic applications.

### 3.5 Biomimetic nanovesicles loaded with PEBP1 mRNA reduce VSMCs ferroptosis and apoptosis

**FIGURE 5:**
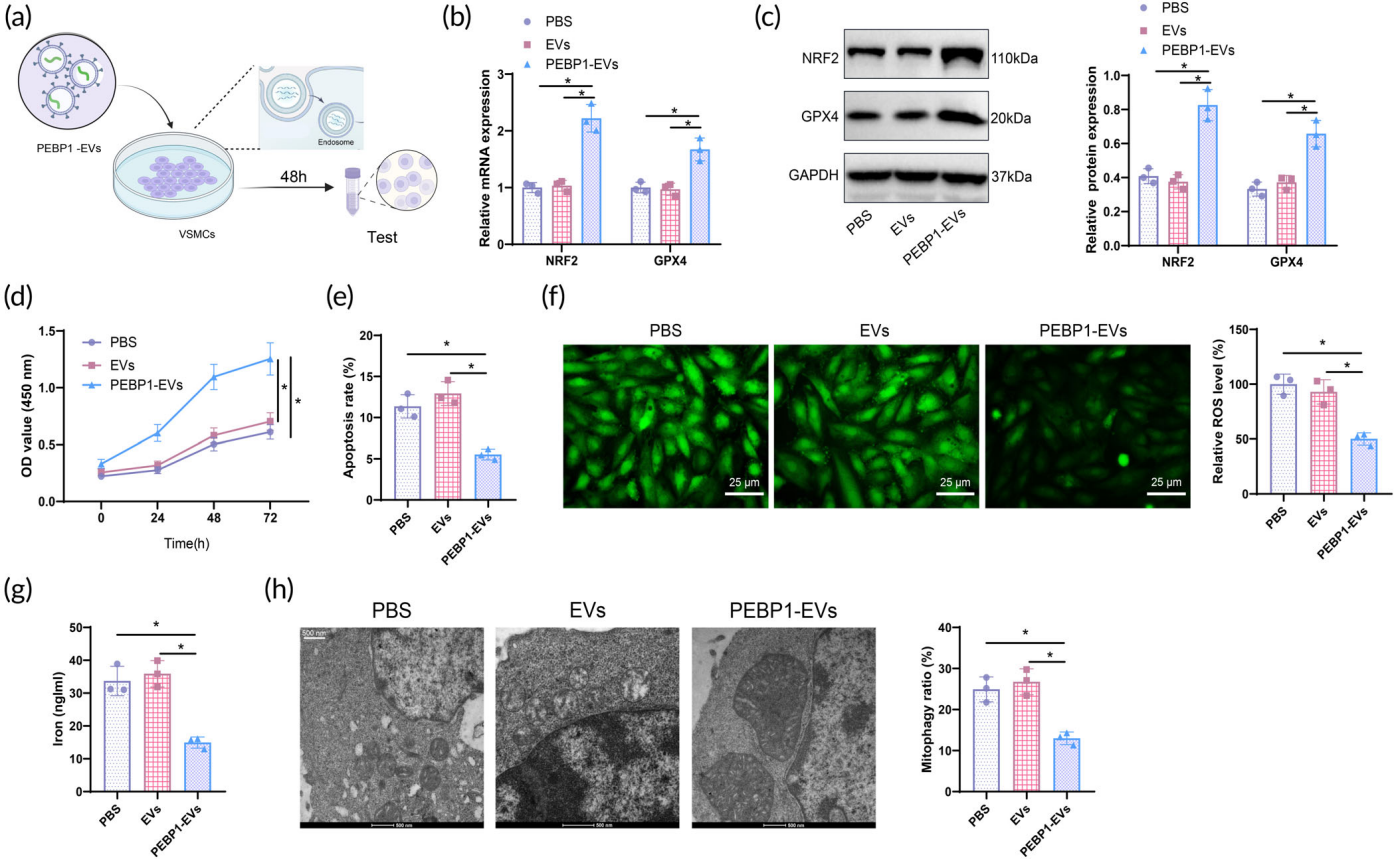
Biomimetic nanovesicles loaded with PEBP1 mRNA modulate ferroptosis and proliferation apoptosis in VSMCs. (a) Diagram of biomimetic nanovesicle treatment of VSMCs. (b) RT‐qPCR analysis of NRF2 and GPX4 mRNA expression in VSMCs from each group. (c) WB analysis of NRF2 and GPX4 protein expression in VSMCs from each group. (d) CCK‐8 assay to assess proliferation capacity of VSMCs. (e) Flow cytometry to determine apoptosis rate of VSMCs. (f) Fluorescent probe DCFH‐DA to measure ROS levels in VSMCs (scale bar: 25 μm). (g) Reagent kit to detect iron ion levels in VSMCs from each group. (h) TEM to observe cellular ultrastructure features in VSMCs from each group (scale bar: 500 nm). *p < 0.05 compared to the PBS group, and cell experiments were repeated three times. Multiple comparisons were conducted using one‐way ANOVA, while different time points' cell viability was analyzed using two‐way ANOVA.

VSMCs were subjected to treatment with biomimetic nanovesicles to evaluate their cellular uptake and impact on the expression of PEBP1/NRF2/GPX4 genes (Figure 5a). Analysis through RT‐qPCR and WB confirmed a significant increase in NRF2 and GPX4 mRNA and protein expression in VSMCs following treatment with nanovesicles loaded with PEBP1 mRNA (Figure 5b,c).

The proliferation capacity of VSMCs was assessed using a CCK‐8 assay, while flow cytometry was employed to determine the apoptosis rate. Results indicated a significant improvement in cell proliferation capacity (Figure 5d) and a notable decrease in the apoptosis rate (Figure 5e) in VSMCs treated with PEBP1 mRNA nanovesicles. Further analysis of the intracellular environment revealed that treatment with PEBP1 mRNA nanovesicles significantly decreased ROS and iron ion levels in VSMCs (Figure 5f,g). Electron microscopy observations further showed that after treatment with PEBP1 mRNA nanovesicles, the mitochondrial structure of VSMCs remained intact, and the phenomenon of cell membrane rupture significantly decreased (Figure 5h). These findings suggest that nanovesicles, by delivering PEBP1 mRNA, effectively activate the NRF2/GPX4 axis, reducing intracellular oxidative stress and iron ion toxicity and potentially attenuating VSMC damage associated with AAA.

### 3.6 Biomimetic nanovesicles loaded with PEBP1 mRNA significantly inhibit the progression of AAA in mice

**FIGURE 6:**
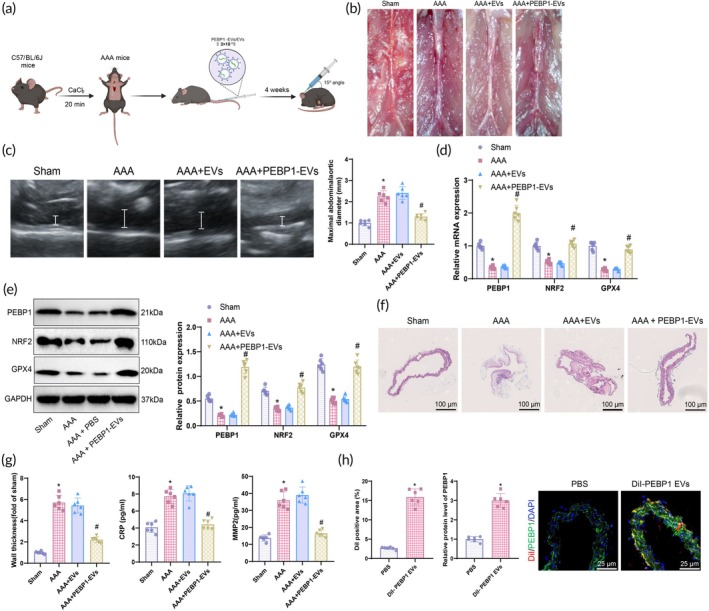
Biomimetic nanovesicles loaded with PEBP1 mRNA significantly attenuate the progression of AAA in mice. (a) Diagram showing the significant inhibition of AAA in mice by PEBP1 mRNA‐loaded nanovesicle treatment. (b) Morphology of AAA in mice from each group (scale bar: 2 mm). (c) Ultrasonography assessment of the morphology and average diameter of aortic aneurysm in mice from each group (white lines indicate abdominal aorta). (d) RT‐qPCR analysis of PEBP1, NRF2, and GPX4 mRNA expression levels in abdominal aortic tissues of mice from each group. (e) WB analysis of PEBP1, NRF2, and GPX4 protein levels in abdominal aortic tissues of mice from each group. (f) H&E staining to examine pathological changes in abdominal aortic tissues of mice from each group (scale bar: 100 μm). (g) ELISA to measure levels of CRP and MMP2 in the serum of mice from each group. (h) Immunofluorescence detection of the homing of biomimetic nanovesicles in the aortic wall (scale bar: 25 μm). On the right side, quantification of EVs and PEBP1 expression. *p < 0.05 compared to the sham/PBS group, #*p* < 0.05 compared to the AAA + PBS group. The two‐group comparisons were analyzed using independent samples *t*‐tests; multiple group comparisons were performed using one‐way ANOVA, with six animals in each group.

The aforementioned in vitro experiments confirm the successful construction of biomimetic nanovesicles loaded with PEBP1 mRNA. By treating VSMC cells, these nanovesicles can inhibit the accumulation of ROS and iron ions during the process of ferroptosis (Figure 6a), thereby protecting the cells from ferroptosis‐induced damage. To further verify the impact of these biomimetic nanovesicles loaded with PEBP1 mRNA on AAA in vivo, we established an in situ model of AAA in mice and applied biomimetic nanovesicles for treatment, achieving a significant therapeutic effect.

Through tail vein injection of nanovesicles loaded with PEBP1 mRNA, the mouse model showed, morphologically and with ultrasound detection, that compared to the Sham group, the average diameter of AAA in the mice increased significantly. After 4 weeks of treatment, the average diameter of the aneurysm decreased compared to pre‐treatment, and the shape became more regular, indicating that the nanovesicles effectively inhibited the progression of the aneurysm (Figure 6b,c).

At the molecular level, RT‐qPCR and WB results indicate that compared to the Sham group, the expression levels of PEBP1 mRNA, NRF2, and GPX4 proteins in the abdominal aortic tissues of mice in the AAA group were significantly decreased. Conversely, compared to the AAA + EVs group, the expression levels of PEBP1 mRNA, NRF2, and GPX4 proteins in the abdominal aortic tissues of mice in the AAA + PEBP1‐EVs group were significantly higher than those in the control group (Figure 6d,e). These findings suggest that the successful expression of PEBP1 mRNA activates the NRF2/GPX4 signaling axis, potentially inhibiting the further development of the aneurysm through this mechanism.

Furthermore, the therapeutic effects were further validated through histological analysis and detection of serum biomarkers. H&E staining of the abdominal aortic tissues revealed that compared to the Sham group, mice in the AAA group exhibited inflammation, tissue damage, and increased vascular wall thickness. In contrast, the treatment group showed a more intact arterial wall structure, significantly reduced inflammation and tissue damage, and decreased vascular wall thickness (Figure 6f). ELISA analysis of serum levels of CRP and MMP2 in the mice from each group showed that compared to the Sham group, mice in the AAA group had significantly elevated levels of CRP and MMP2. Moreover, compared to the AAA + EVs group, mice in the AAA + PEBP1‐EVs group displayed significantly lower levels of CRP and MMP2 in the serum (Figure 6g). Finally, we studied the homing location of biomimetic nanovesicles in the mouse aortic wall using immunofluorescence. The immunofluorescence images confirmed that DiI‐PEBP1 EVs were present in the aortic wall, indicating that DiI‐PEBP1 EVs were effectively absorbed by the aortic wall. Compared to the PBS group, the PEBP1 protein expression was higher in the DiI‐PEBP1 EVs group, suggesting that PEBP1 mRNA was successfully expressed after internalization of PEBP1 EVs into the aortic wall (Figure 6h).

In conclusion, the above results further demonstrate that PEBP1‐EVs can inhibit the NRF2/GPX4 axis, thereby alleviating the development of AAA.

## 4 DISCUSSION

This study utilized biomimetic nanovesicles loaded with PEBP1 mRNA to activate the NRF2/GPX4 axis and suppress ferroptosis in VSMCs, aiming to prevent the formation of AAA. The research not only elucidates the significant role of ferroptosis in the pathogenesis of AAA but also proposes a novel molecular‐targeted therapeutic approach. Our key findings demonstrate that overexpression of PEBP1 can promote VSMC proliferation, inhibit intracellular ROS and iron levels, decrease cell apoptosis, and prevent ferroptosis by activating the NRF2/GPX4 axis. This innovative research avenue offers a potential molecular targeted strategy for the prevention and treatment of AAA.

Ferroptosis, a form of iron‐dependent cell death, has garnered increasing attention in the study of AAA in recent years.^23^, ^24^, ^25^ Prior research has indicated the significant role of ferroptosis in atherosclerosis and other vascular disorders; however, its specific mechanisms in AAA remain unclear.^62^, ^63^, ^64^ Our study, through data analysis from the GEO database and FerrDb database, identified 12 ferroptosis‐related genes with altered expression in AAA, further confirming PEBP1 as a pivotal gene among them. This discovery refines the molecular mechanisms of ferroptosis in AAA compared to previous studies, particularly emphasizing the unique role of PEBP1 in ferroptosis.

The critical regulatory role of the NRF2/GPX4 axis in cellular antioxidant stress response and ferroptosis has been widely recognized.^27^, ^28^, ^29^ NRF2 induces the expression of antioxidant genes, while GPX4 protects cells from ferroptosis by reducing the accumulation of lipid peroxides. Previous studies have confirmed the protective function of the NRF2/GPX4 axis in various cardiovascular diseases. However, this study is the first to reveal the specific mechanism by which PEBP1 inhibits VSMCs ferroptosis through activating the NRF2/GPX4 axis. This finding not only advances the understanding of the regulatory network of the NRF2/GPX4 axis but also provides a new target for molecular therapy of AAA.

Biomimetic nanovesicles, as a novel drug delivery system, exhibit excellent biocompatibility and targeting properties.^40^, ^41^ Compared to conventional drug delivery systems, nanovesicles can more precisely deliver drugs to target cells or tissues, reducing side effects and enhancing therapeutic outcomes.^40^, ^43^, ^65^ In this study, we successfully synthesized biomimetic nanovesicles loaded with PEBP1 mRNA and validated their biological effects and therapeutic potential through in vitro and in vivo experiments. The results demonstrate that the nanovesicles not only efficiently deliver PEBP1 mRNA but also significantly inhibit ferroptosis in VSMCs and the progression of AAA. This technology offers a new approach and method for the treatment of AAA.

The results of both in vitro and in vivo experiments mutually corroborate, further supporting the effectiveness of PEBP1 mRNA in nanovesicles. In the in vitro experiments, we observed successful expression of PEBP1 mRNA and activation of the NRF2/GPX4 axis, leading to the inhibition of ferroptosis and apoptosis in VSMCs. Additionally, the expression of the PEBP1/NRF2/GPX4 axis was verified through techniques such as fluorescence confocal microscopy, RT‐qPCR, and WB analysis. In the in vivo experiments, nanovesicle treatment significantly suppressed the progression of AAA in mice, a conclusion supported by both ultrasound detection and histological analysis. Compared to previous studies, the experimental design in this study is more rigorous, the data is more reliable, and the results are more persuasive.

Compared to other molecular targeting therapeutic strategies, the PEBP1/NRF2/GPX4 axis activation strategy proposed in this study offers significant advantages. Prior treatment strategies for AAA have mainly focused on aspects such as anti‐inflammatory, antioxidant, and matrix remodeling. However, this study, through the inhibition of ferroptosis, introduces a new pathway for AAA treatment. Furthermore, the application of biomimetic nanovesicles as a drug delivery system enhances the targeting and effectiveness of treatment. Our research findings indicate that the PEBP1/NRF2/GPX4 axis activation strategy is not only effective under laboratory conditions but also holds promise for significant clinical impact in the future.

**FIGURE 7:**
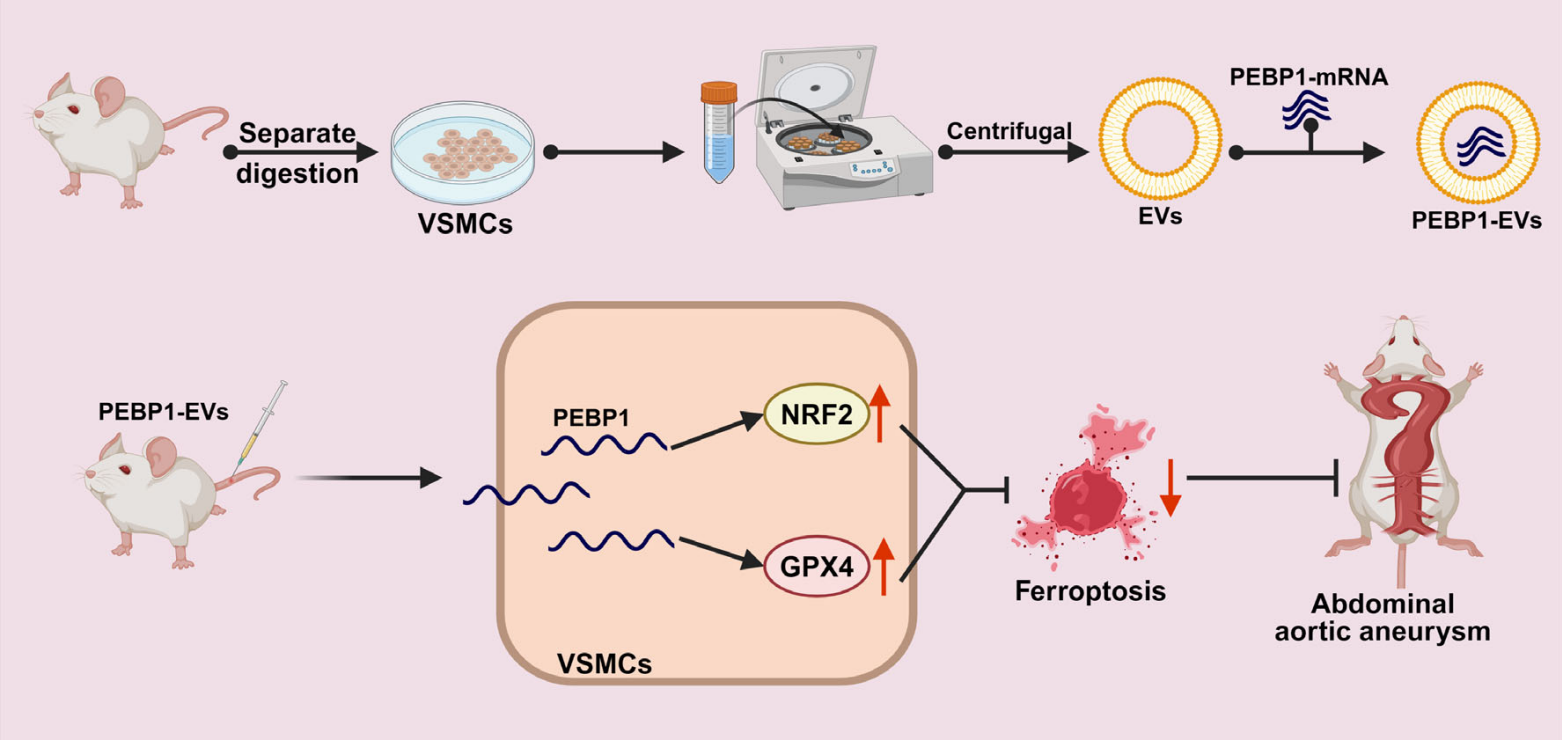
Molecular mechanism diagram of PEBP1‐EVs activating the NRF2/GPX4 axis in anti‐ferroptosis pathway.

This study discovered that biomimetic nanovesicles loaded with PEBP1 mRNA effectively activate the NRF2/GPX4 axis, inhibiting ferroptosis in VSMCs, thereby preventing the formation of AAA. This novel treatment provides a potential molecular targeting strategy for the prevention and treatment of AAA (Figure 7). Experimental results demonstrate that PEBP1 overexpression activates the NRF2/GPX4 axis, suppressing ROS and iron ion levels, thus reducing ferroptosis in VSMCs. Subsequently, nanovesicles carrying PEBP1 mRNA were successfully synthesized, and these nanovesicles demonstrated the ability to reduce VSMCs' ferroptosis by delivering PEBP1 mRNA. Finally, in vivo animal experiments confirmed that nanovesicle treatment significantly inhibits the progression of AAA in mice.

This study innovatively applied biomimetic nanovesicles loaded with PEBP1 mRNA to elucidate the crucial role of the NRF2/GPX4 axis in inhibiting ferroptosis in VSMCs and preventing the formation of AAA. This finding not only enhances our understanding of the mechanism of ferroptosis but also offers a novel direction for molecular targeted therapy of AAAs. Through in vivo and in vitro experiments, we demonstrated that PEBP1 mRNA efficiently expresses and exerts a protective effect in nanovesicles, providing a potential molecular target for AAA treatment. Clinically, this research lays the foundation for developing efficient and highly targeted novel therapeutic strategies, which could be validated in clinical trials in the future to prove their effectiveness and safety in the prevention and treatment of AAAs.

While this study has made significant progress, some limitations must be acknowledged. Firstly, the small sample size may restrict the generalizability and statistical significance of the results. Secondly, the experimental models primarily focused on animal studies, necessitating further preclinical research and clinical trials to validate the efficacy and safety in human patients. Additionally, the long‐term biocompatibility and stability of nanovesicles have not been fully assessed, requiring more studies to ensure their feasibility and safety in clinical applications. Additionally, this study primarily investigated the role of the PEBP1/NRF2/GPX4 axis in VSMCs, and future research should explore the applicability and mechanisms of this pathway in other cell types and vascular diseases. Finally, this study only used male mice, which may affect the generalizability of the results, particularly regarding the impact of sex differences on treatment response. Future studies should include both male and female mice to explore potential sex differences and their influence on the findings.

Future research should focus on increasing sample sizes and diversifying experimental models to validate the universality and reliability of the findings of this study. Further optimization of the design of nanovesicles to enhance their stability and biocompatibility may promote their clinical application. Exploring the mechanisms of the PEBP1/NRF2/GPX4 axis in other vascular diseases may reveal additional therapeutic potential and application prospects. By conducting multicenter clinical trials to evaluate the effectiveness and safety of biomimetic nanovesicles loaded with PEBP1 mRNA in AAA patients, the translation from experimental research to clinical application can be achieved. Additionally, combining other molecular‐targeted therapy strategies to develop comprehensive treatment plans holds the promise of further improving the prevention and treatment outcomes of AAAs.

## AUTHOR CONTRIBUTIONS

Lulu Chen designed and conducted the experiments, performed data analysis, and drafted the manuscript. Bicheng Chen contributed to the conceptualization of the study, supervised the research, and provided critical revisions to the manuscript. Xiang Su led the in vivo studies, analyzed single‐cell data, and provided expertise in vascular surgery and ferroptosis mechanisms. All authors contributed to the final manuscript, read, and approved the submitted version.

## CONFLICT OF INTEREST STATEMENT

The authors declare no conflicts of interest.

## Supporting information


**FIGURE S1:** Quality control, filtering, and PCA of scRNA‐seq data. (a) Violin plots depicting the gene counts (nFeature_RNA), mRNA molecule counts (nCount_RNA), and mitochondrial gene percentage (percent.mt) for each cell in the scRNA‐seq dataset. (b) Scatter plots showing the correlation between filtered data nCount_RNA and percent.mt, as well as nCount_RNA and nFeature_RNA. (c) Heatmap displaying the top 20 significantly correlated gene expressions in PC_1–PC_6 of PCA, where yellow represents upregulated expression and purple represents downregulated expression. (d) Distribution of cells in PC_1 and PC_2 before batch correction on the left, with each point representing a cell, and violin plot of the distribution in PC_1 and PC_2 on the right. (e) Illustration of the batch correction process using Harmony, with the number of interaction cycles on the *x*‐axis. (f) Distribution of standard deviations of PCs, where important PCs exhibit larger standard deviations. AAA: *n* = 4.

**FIGURE S2:** Cell clustering of scRNA‐seq data. (a) Left: Distribution of cells in PC_1 and PC_2 after Harmony batch correction, with each point representing a cell; Right: Violin plot after correction. (b) Clustree package showing clustering results at different resolutions. (c) Visualization of UMAP clustering results depicting the clustering and distribution of cells, with each color representing a cluster. AAA: *n* = 4.

**FIGURE S3:** Expression of the specific marker α‐SMA in VSMCs and optimization of lentiviral transfection conditions. (a) Immunofluorescence detection of α‐SMA expression in primary VSMCs (scale bar: 25 μm). (b) Detection of PEBP1 expression after lentiviral transfection at different doses. (c) Detection of PEBP1 expression after lentiviral transfection for different time points. *p < 0.05. Multiple groups were analyzed using one‐way ANOVA, and cell experiments were repeated three times.

**FIGURE S4:** Preferential uptake of EVs by VSMCs and EV dose‐dependent experiment. (a) Immunofluorescence of VSMCs/HEK293T cells co‐cultured with VSMC‐derived EVs (scale bar: 15 μm). (b) RT‐qPCR detection of PEBP1 mRNA expression in HEK293T and VSMCs in each group. (c) WB detection of PEBP1 protein expression in HEK293T and VSMCs in each group. (d) Immunofluorescence and quantification of PEBP1 after EV treatment at different doses (scale bar: 25 μm). *p < 0.05. The two‐group comparisons were analyzed using independent samples *t*‐tests; multiple group comparisons were performed using one‐way ANOVA, and cell experiments were repeated three times.

## Data Availability

The data that support the findings of this study are available from the corresponding author upon reasonable request.
