# Supplementary figures and images for: Engineering biomimetic nanovesicles for PEBP1 mRNA delivery to inhibit ferroptosis in abdominal aortic aneurysm
Source: Bioeng Transl Med. 2025 Jul 21;10(5):e70025. doi: 10.1002/btm2.70025 (PMC12478448; doi:10.1002/btm2.70025)

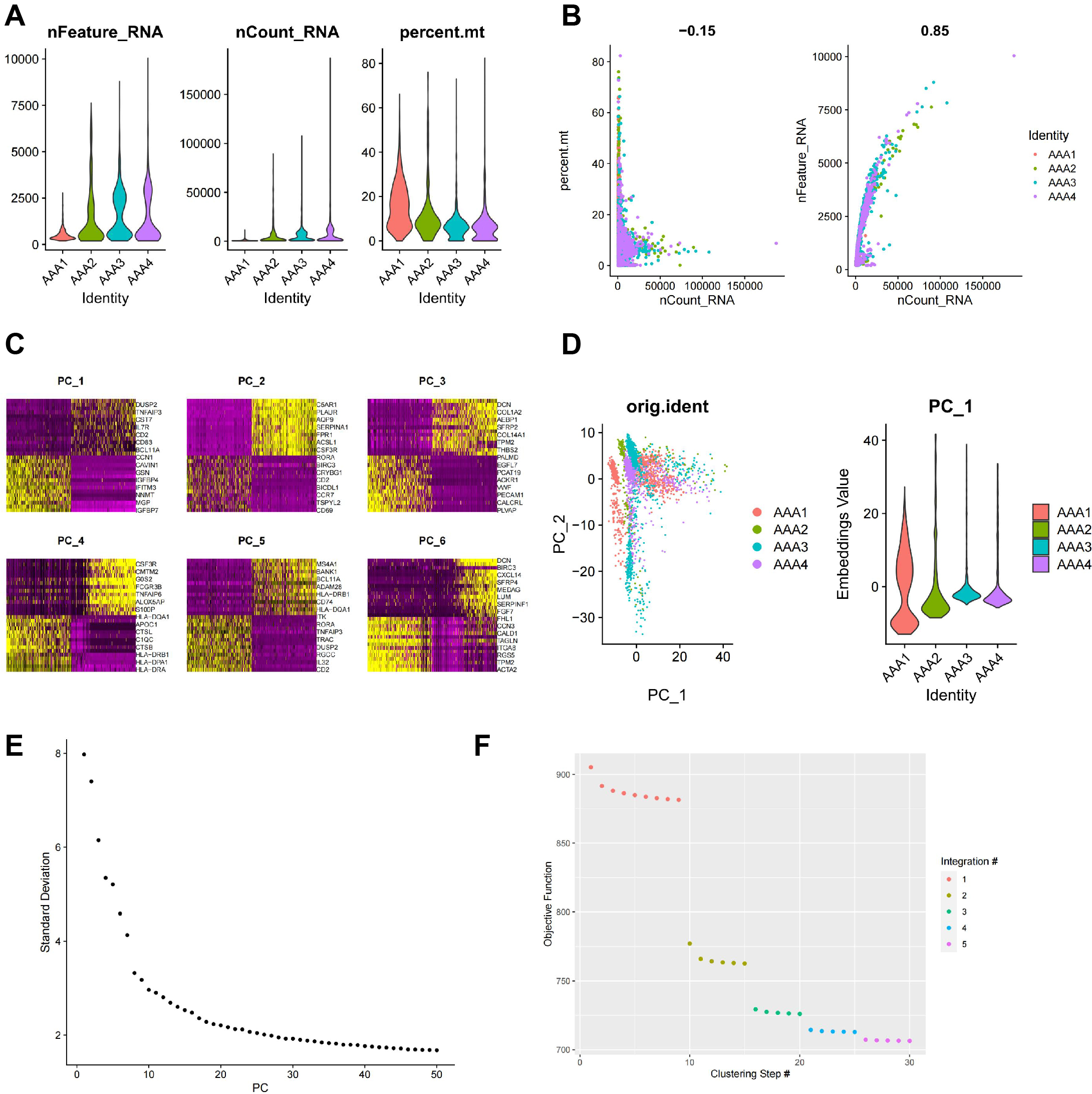

Supplement: Supplementary file 1 — FIGURE S1: Quality control, filtering, and PCA of scRNA‐seq data. (a) Violin plots depicting the gene counts (nFeature_RNA), mRNA molecule counts (nCount_RNA), and mitochondrial gene percentage (percent.mt) for each cell in the scRNA‐seq dataset. (b) Scatter plots showing the correlation between filtered data nCount_RNA and percent.mt, as well as nCount_RNA and nFeature_RNA. (c) Heatmap displaying the top 20 significantly correlated gene expressions in PC_1–PC_6 of PCA, where yellow represents upregulated expression and purple represents downregulated expression. (d) Distribution of cells in PC_1 and PC_2 before batch correction on the left, with each point representing a cell, and violin plot of the distribution in PC_1 and PC_2 on the right. (e) Illustration of the batch correction process using Harmony, with the number of interaction cycles on the x‐axis. (f) Distribution of standard deviations of PCs, where important PCs exhibit larger standard deviations. AAA: n = 4. [file BTM2-10-e70025-s002.jpg]

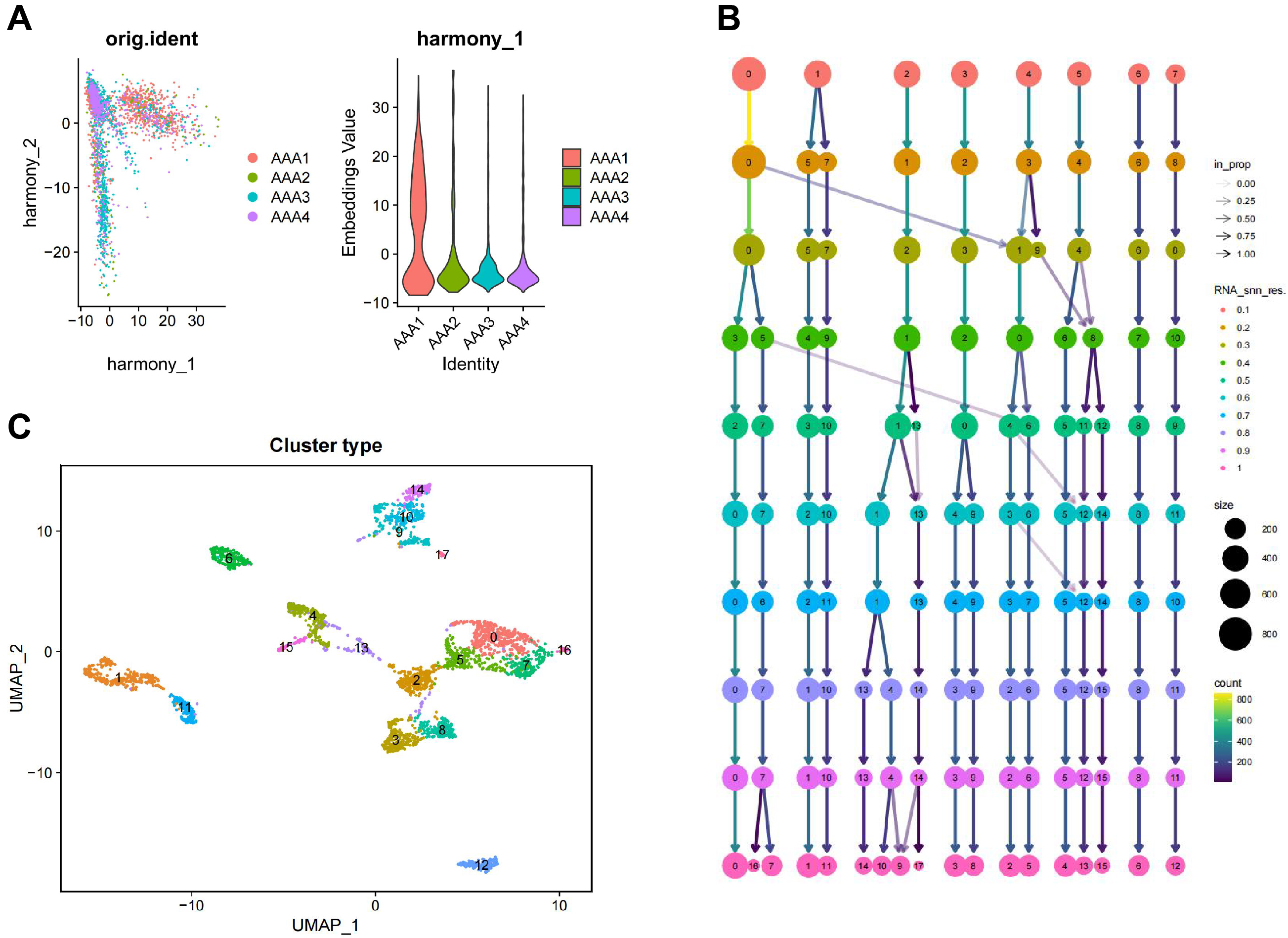

Supplement: Supplementary file 2 — FIGURE S2: Cell clustering of scRNA‐seq data. (a) Left: Distribution of cells in PC_1 and PC_2 after Harmony batch correction, with each point representing a cell; Right: Violin plot after correction. (b) Clustree package showing clustering results at different resolutions. (c) Visualization of UMAP clustering results depicting the clustering and distribution of cells, with each color representing a cluster. AAA: n = 4. [file BTM2-10-e70025-s004.jpg]

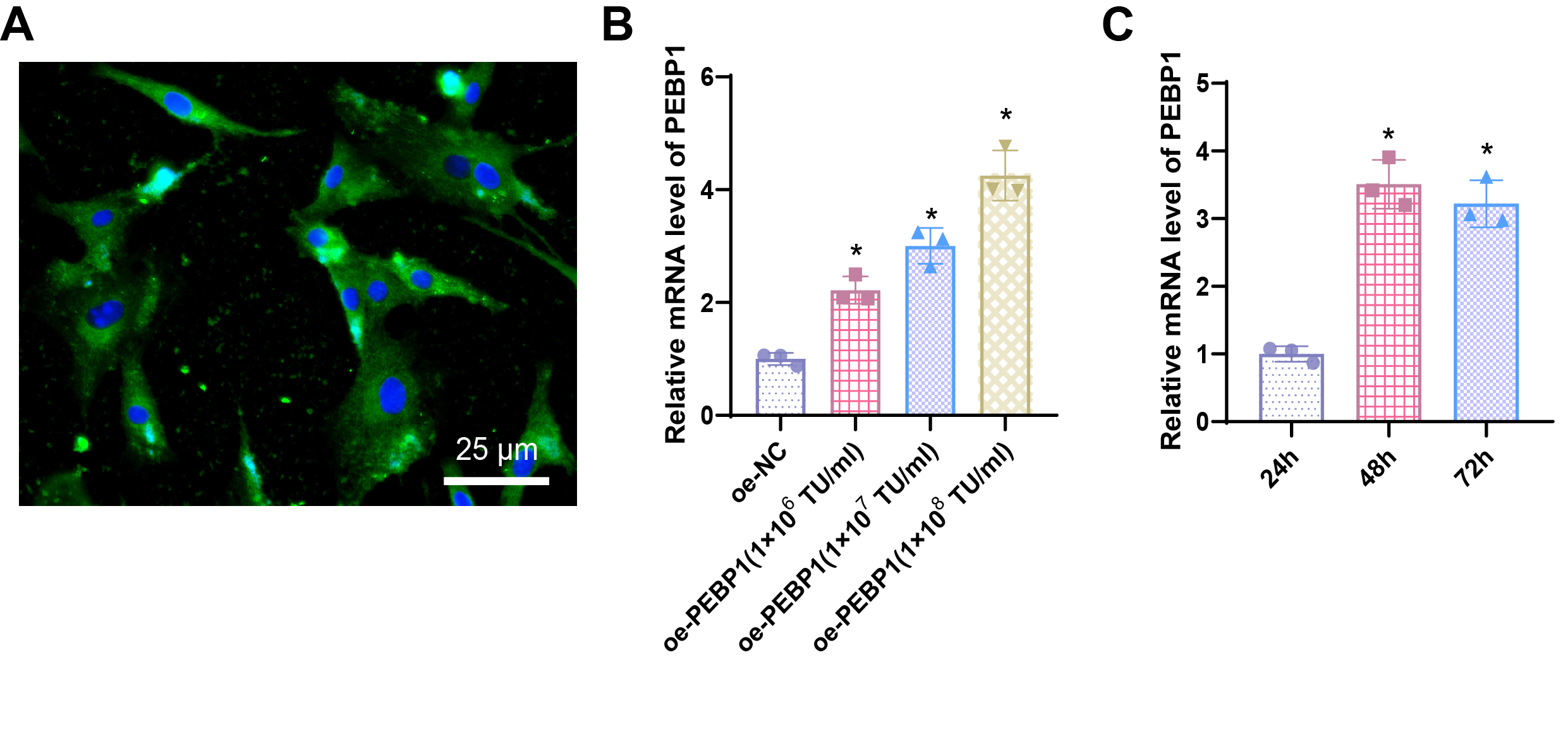

Supplement: Supplementary file 3 — FIGURE S3: Expression of the specific marker α‐SMA in VSMCs and optimization of lentiviral transfection conditions. (a) Immunofluorescence detection of α‐SMA expression in primary VSMCs (scale bar: 25 μm). (b) Detection of PEBP1 expression after lentiviral transfection at different doses. (c) Detection of PEBP1 expression after lentiviral transfection for different time points. *p < 0.05. Multiple groups were analyzed using one‐way ANOVA, and cell experiments were repeated three times. [file BTM2-10-e70025-s001.jpg]

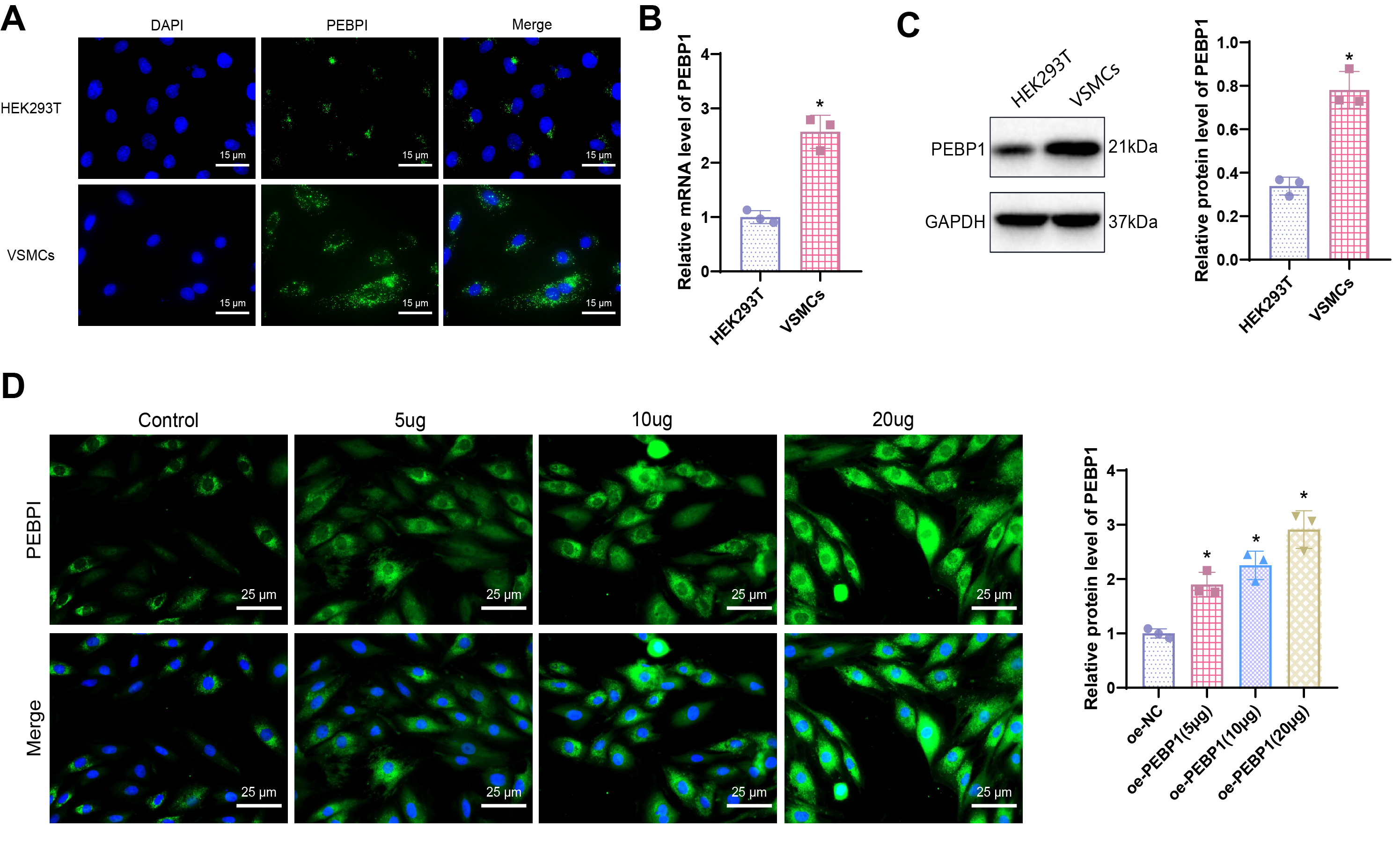

Supplement: Supplementary file 4 — FIGURE S4: Preferential uptake of EVs by VSMCs and EV dose‐dependent experiment. (a) Immunofluorescence of VSMCs/HEK293T cells co‐cultured with VSMC‐derived EVs (scale bar: 15 μm). (b) RT‐qPCR detection of PEBP1 mRNA expression in HEK293T and VSMCs in each group. (c) WB detection of PEBP1 protein expression in HEK293T and VSMCs in each group. (d) Immunofluorescence and quantification of PEBP1 after EV treatment at different doses (scale bar: 25 μm). *p < 0.05. The two‐group comparisons were analyzed using independent samples t‐tests; multiple group comparisons were performed using one‐way ANOVA, and cell experiments were repeated three times. [file BTM2-10-e70025-s003.jpg]
